# Supplementary material for: Tellurium-Doped, Mesoporous Carbon Nanomaterials as Transparent Metal-Free Counter Electrodes for High-Performance Bifacial Dye-Sensitized Solar Cells
Source: Nanomaterials (Basel). 2019 Dec 20;10(1):29. doi: 10.3390/nano10010029 (PMC7022714; doi:10.3390/nano10010029)
Supplement: Supplementary file 1 [file nanomaterials-10-00029-s001.pdf]

Supporting Information

Tellurium-Doped Mesoporous Carbon Nanomaterials as Transparent Metal-Free Counter Electrodes for High Performance Bifacial Dye-Sensitized Solar Cells

Chang Ki Kim, Jung-Min Ji, Haoran Zhou, Chunyuan Lu and Hwan Kyu Kim *

Global GET-Future Lab. & Department of Advanced Materials Chemistry, Korea University 2511 Sejong-ro, Sejong 339-700, Korea

* Correspondence: hkk777@korea.ac.kr

Characterization. Field emission scanning electron microscopy (FE-SEM) was measured with a S-4700 (Hitachi, Japan) microscope to confirm the surface morphology of the prepared materials and electrodes. Transmission electron microscopy (TEM) was performed on an EM 912 Omega microscope at 120 kV. X-ray photoelectron spectroscopy (XPS) was carried out with an AXIS-NOVA (Kratos) using an Al-Kα X-ray source. X-ray diffraction (XRD) was performed on a Rigaku Smart Lab diffractometer with CuKα radiation measured at 40 kV and 30 mA. The nitrogen sorption isotherms were measured by Micromeritics ASAP 2020 equipment. Specific surface areas of TeMC materials were calculated by the Brunauer-Emmett-Teller (BET) equation using the nitrogen adsorption data. Total pore volumes were determined by the amount of gas adsorbed at a relative pressure of 0.99. Pore size distributions were calculated by Micromeritics software based on the density functional theory method.

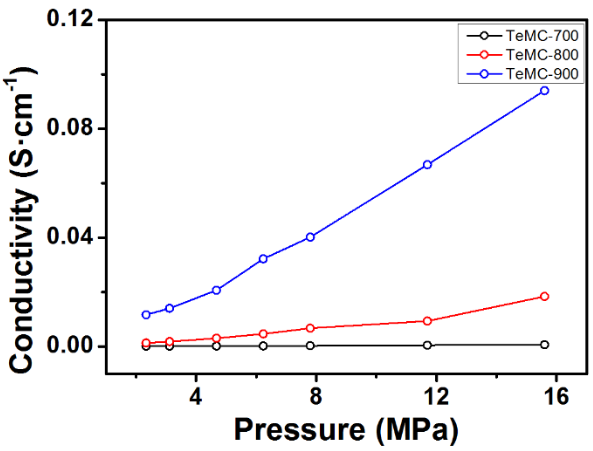

Figure S1. Electrical conductivity data of TeMC materials with different carbonization temperatures.

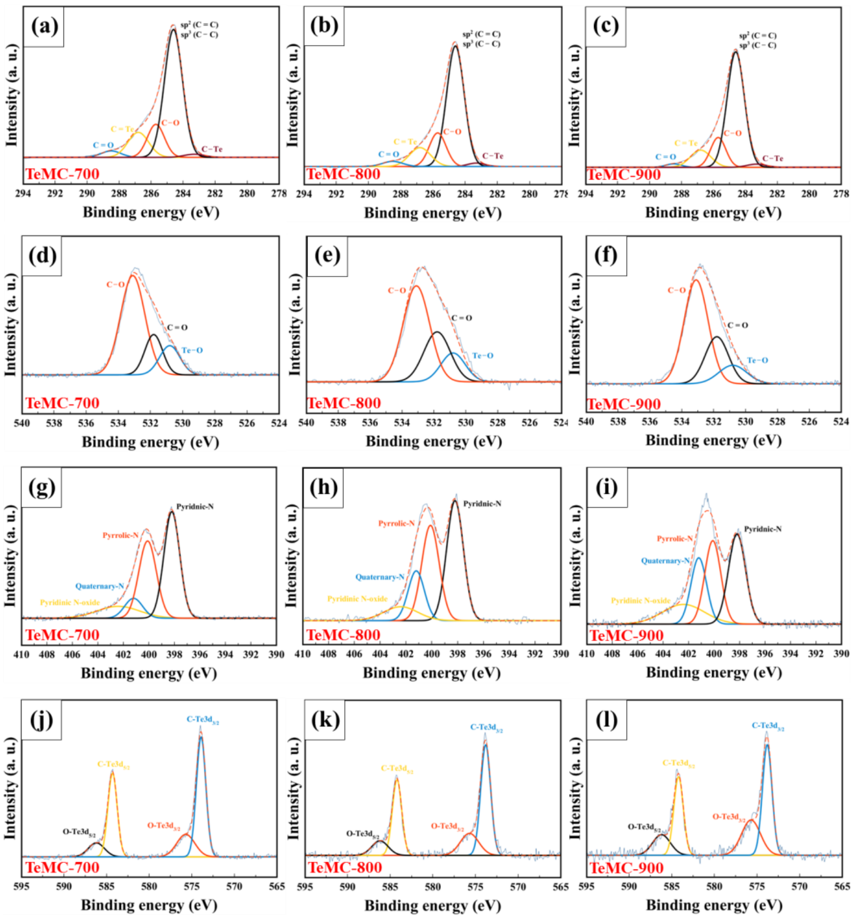

Figure S2. High-resolution XPS spectra of TeMC materials with different carbonization temperatures (**a,b,c**: carbon, **d,e,f**: oxygen, **g,h,i**: nitrogen, **j,k,l**: tellurium).

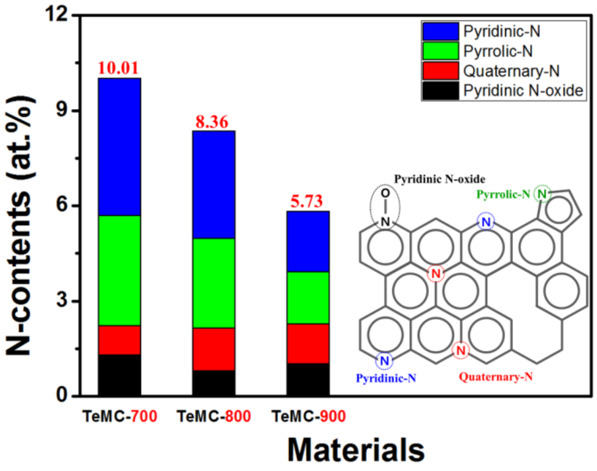

Fig. S3. Bonding distributions of nitrogen in TeMC materials. The inset is the bonding configurations for nitrogen.

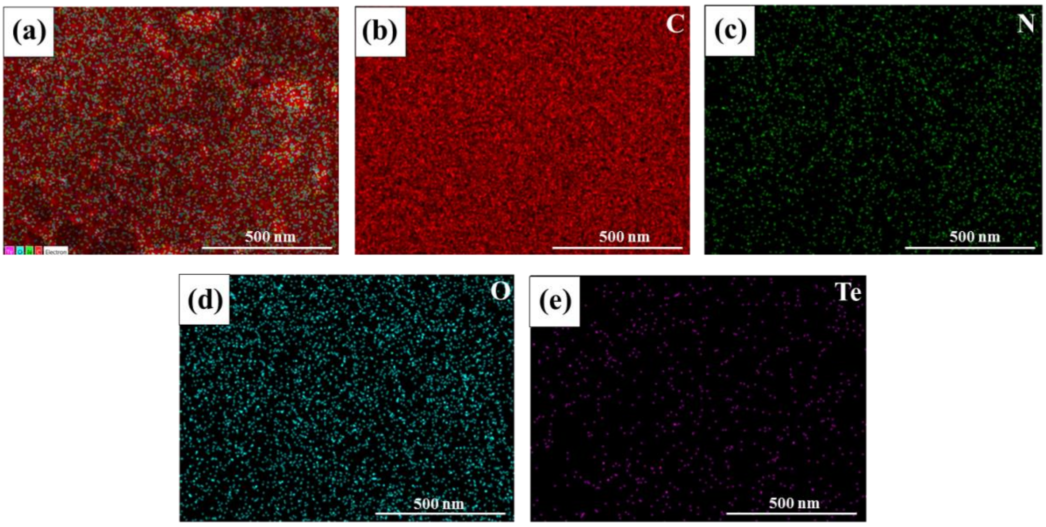

Fig. S4. (**a**) SEM image and (**b–e**) corresponding elemental mappings of TeMC materials (**b**: carbon, **c**: nitrogen, **d**: oxygen, **e**: tellurium).

Preparation of TeMC electrodes. About 0.1 wt% of TeMC materials was dispersed in isopropyl alcohol solution using ultrasonication. The solution was centrifuged at 3000 rpm for 15 min to remove large particles of TeMC materials. The homogenous solution was directly spread onto FTO glass (TEC-8, Pilkington) using the electrospray method. First, the homogeneous solution was placed in a plastic syringe with a stainless-steel needle (30-gauge). The needle was connected to a high-voltage power supply (ESN-HV30). A voltage of 4.2 kV was applied between the needle and the collector at a distance of 4 cm. The injection rate of the solution was 40 μL min^{-1}, controlled by a syringe pump (KD Scientific Model 220). The electric fields overcome the surface tension of the droplets, resulting in minimization of numerous charged mist particles. The TeMC electrodes were sintered at 280°C for 15 min in air atmosphere before device fabrication. The Pt-FTO electrode (reference electrode) was prepared by deposition of *ca*. 30 μL cm^{-2} of H_2PtCl_6 solution (2 mg of H_2PtCl_6 in 1 mL of ethanol), and it was sintered at 400°C for 15 min.

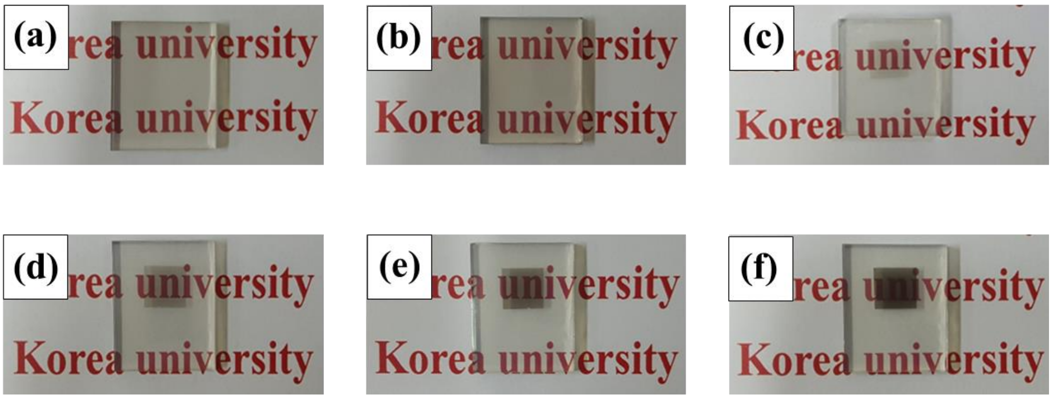

Fig. S5. Digital photograph images of (**a**) FTO glass, (**b**) the Pt CE, and (**c–f**) TeMC CEs (**c**: 0.1, **d**: 0.2, **e**:

0.3 and **f**: 0.5)

Fabrication of symmetrical dummy cell. Symmetrical dummy cells were fabricated with two identical TeMC- and Pt-FTO electrodes, which were separated by 25-μm thick Surlyn (Solaronix, Switzerland) tape as a spacer. The active area of the dummy cell was 0.7 × 0.6 cm. To improve electrical contact, the FTO glass edges were coated with indium using an ultrasonic soldering system (USS-9200, MBR Electronics). $[Co(bpy)_3][B(CN)_4]_2$ (0.22 M), $[Co(bpy)_3][B(CN)_4]_3$ (0.05 M), $LiClO_4$ (0.1 M), and 4-*tert*-butylpyridine (0.8 M) were dissolved in acetonitrile to prepare the cobalt electrolyte. Electrolyte solutions were injected into the space between the two electrodes through a hole in the CEs. The hole was covered by cover glass using a Surlyn seal.

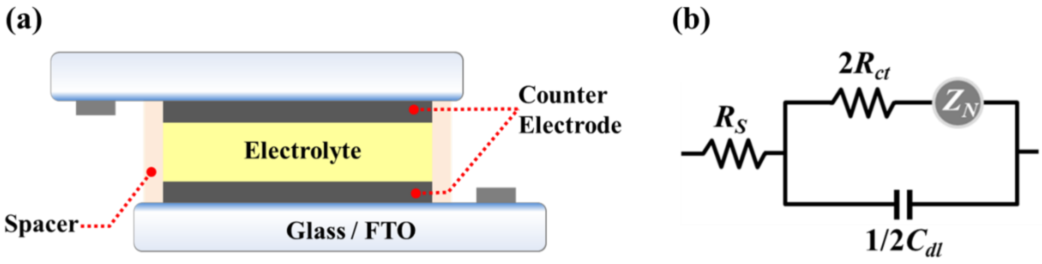

Fig. S6. (**a**) Symmetrical dummy cell and (**b**) the equivalent circuit.

Electrochemical analysis. To evaluate the electrocatalytic activity, electrochemical measurements were carried out using the VersaSTAT 3 (Version 1.31) AMETEK connected to a potentiostat under dark conditions at room temperature. EIS spectra were determined in the frequency range of 10^6 to 10^{-1} Hz at a 0 V open-circuit voltage and AC modulation amplitude of 10 mV. EIS data analysis was fitted using the Zplot/Zview2 software.

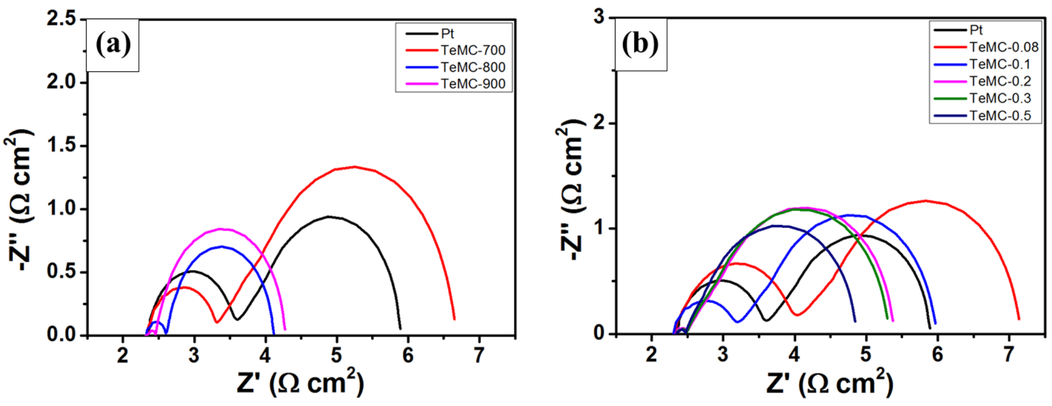

Fig. S7. Nyquist plot of Pt and TeMC CEs with (**a**) different carbonization temperatures and (**b**) various loaded amounts of TeMC materials.

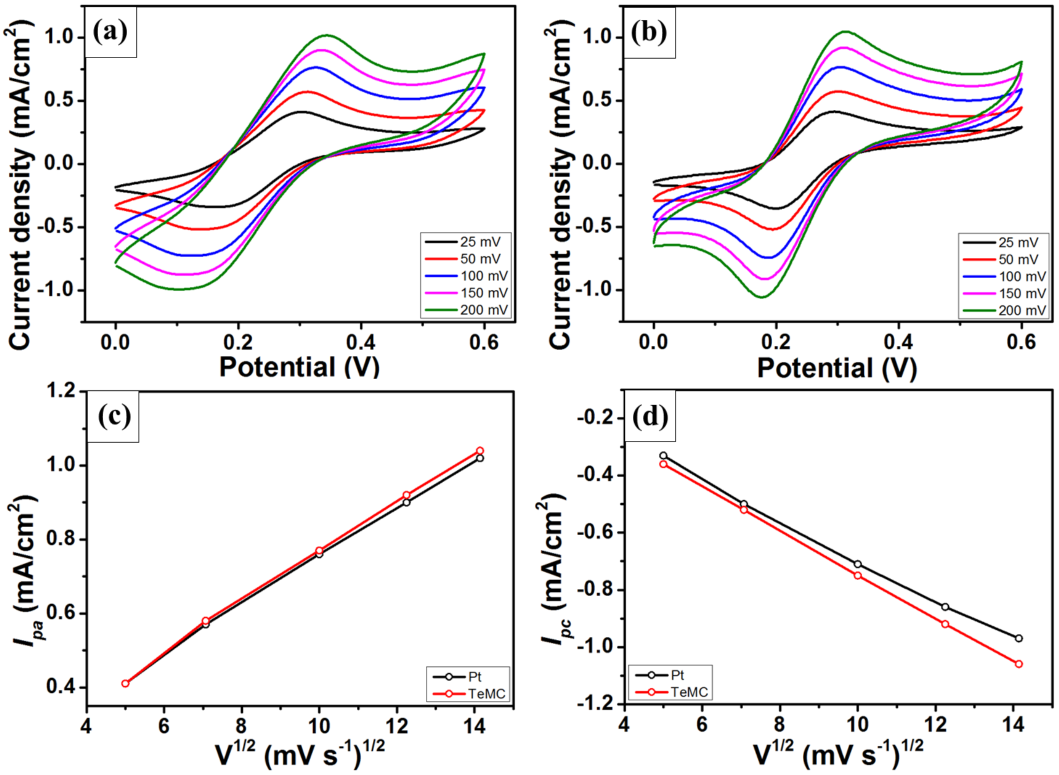

Fig. S8. CV curves of CEs with different scan rates for the cobalt redox couple. (**a**) Pt and (**b**) TeMC electrodes were used as the working electrodes, a Pt wire was the CE, Ag/AgCl was the reference electrode, and 0.1 M $LiClO_4$ was the supporting electrolyte. (**c**) Oxidation and (**d**) reduction peak currents with respect to the square root of the scan rate.

Fabrication and characterization of DSSCs. First, FTO glass substrates were cut to the size of 4.5 × 9.0 cm and cleaned in detergent solution (water/ethanol/acetone, 1:1:1 volume ratio) using an ultrasonic bath. After washing, they were immersed in 40 mM aqueous $TiCl_4$ solution at 70°C for 30 min and washed with water and ethanol. TiO_2 paste (Dyesol, 18NR-T) was screen-printed onto the FTO glass and sintered at 500°C for 30 min in air. The thickness of the TiO_2 active layer (active layer thickness: 4.5 μm) was confirmed by an Alpha Step 250 surface profile meter (Tencor Instruments, San Jose, CA). The scattering layer (scattering layer thickness: 2.5 μm) was deposited by doctor blade printing and then dried at 25°C for 2 h (with scattering layer: DSSCs, without scattering layer: bifacial DSSCs). TiO_2 electrodes were sintered at 500°C for 30 min. The resulting TiO_2 photoanode was immersed in THF/ethanol (v/v, 1:3) solution containing 0.2 mM of sensitizers (SM315) and 0.6 mM of CDCA co-adsorbent, and then kept at room temperature for 2 h. The dye-adsorbed TiO_2 photoanodes were assembled with Pt and TeMC CEs using a thermal adhesive film (25-μm thick Surlyn, DuPont) as a spacer to produce a sandwich-type cell. The electrolyte solution, which was prepared by dissolving $Co(bpy)_3[B(CN)_4]_2$ (0.22 M), $Co(bpy)_3[B(CN)_4]_3$ (0.05 M), $LiClO_4$ (0.1 M), and 4-tert-butylpyridine (0.8 M) in acetonitrile, was injected through a hole in the CE. The hole was covered by a piece of cover glass using Surlyn. The light intensity was adjusted with a Si solar cell that was doubled-checked with a NREL-calibrated Si solar cell (PV Measurement Inc.). The applied potential and measured cell current were measured with a Keithley model 2400 digital source meter. Photoelectrochemical data were measured with a 1000 W xenon light source (Oriel, 91193) that was focused to give 100 mW cm^{-2} (1 sun at AM 1.5 G). The *J*–*V* characteristics of the cell under these conditions were determined by biasing the cell externally and measuring the generated photocurrent

(A mask with an aperture of 0.141cm^2 is used to define the active area of the test cells). This process was fully automated using Wavemetrics software. The measurement settling time between applying the voltage and measuring the current for the *J*–*V* characterisation of the DSSCs was fixed at 80 ms.

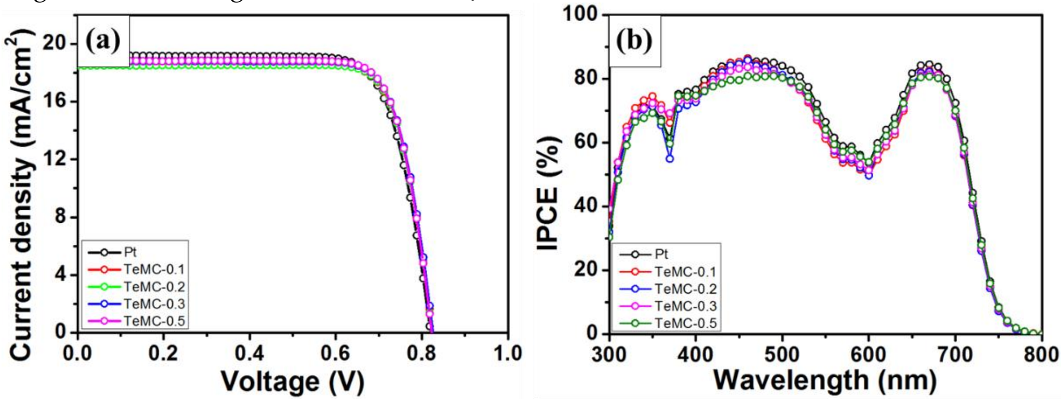

Fig. S9. (**a**) *J*–*V* curves of SM315/Co(bpy)$_3^{2+/3+}$ based DSSC devices with Pt and TeMC CEs (with different loaded amounts of the TeMC CE) under one sun illumination (AM 1.5 G). (**b**) The corresponding IPCE data of DSSC devices.

Table S1. Photovoltaic performance of DSSCs with Pt and TeMC CEs.

Sample	Dye/Electrolyte	J_{SC} [mA cm^{-2}]	V_{OC} [mV]	*FF* [%]	PCE [%]*
Pt		19.00 ± 0.14	830.40 ± 1.13	77.08 ± 0.30	12.01 ± 0.08
**TeMC-0.1**		18.49 ± 0.18	822.31 ± 3.79	79.36 ± 0.29	12.07 ± 0.10
**TeMC-0.2**	**SM315** / Co(bpy)$_3^{2+/3+}$	18.46 ± 0.13	823.89 ± 2.95	79.55 ± 0.38	12.10 ± 0.07
**TeMC-0.3**		18.55 ± 0.24	823.82 ± 1.82	79.39 ± 0.34	12.13 ± 0.12
**TeMC-0.5**		18.56 ± 0.22	824.92 ± 1.77	79.48 ± 0.29	12.17 ± 0.13

*The average power conversion efficiency (PCE) was calculated in five different cells, measured under AM 1.5 G illumination (100 mW cm^{-2}).

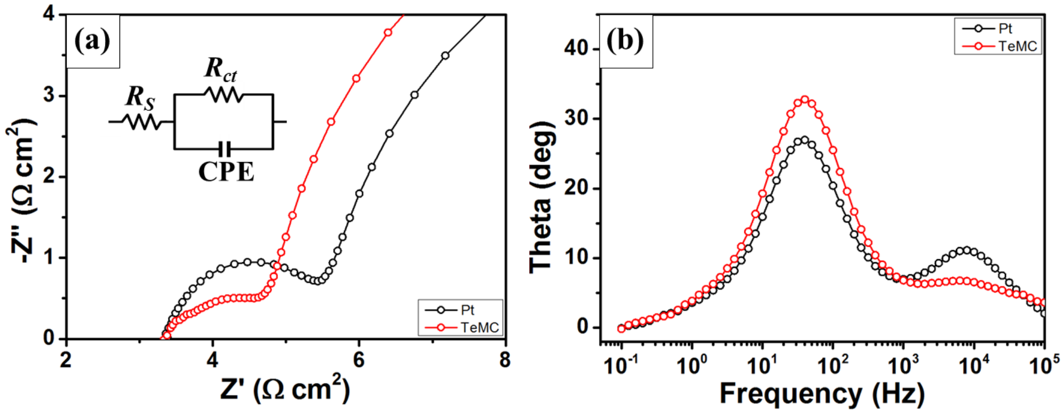

Fig. S10. (**a**) Expended impedance spectra of the high-frequency region for SM315/Co(bpy)$_3^{2+/3+}$ based DSSC devices employing Pt and TeMC CEs. (**b**) Bode plots obtained with the same devices.

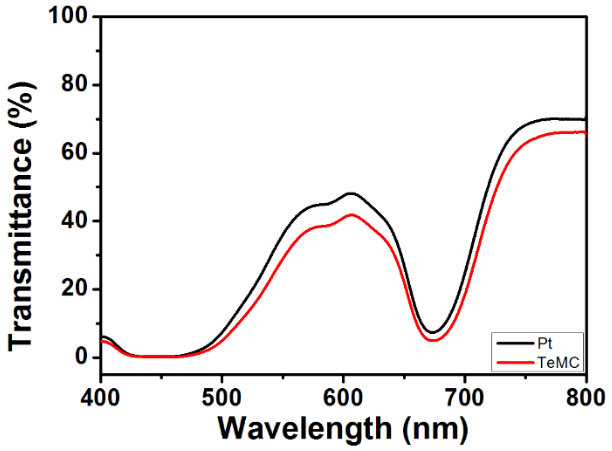

Fig. S11. Transmittances spectra of the DSSC devices with Pt and TeMC CEs

Table S2. Comparison of reported performances in bifacial DSSCs.

CE	Electrolyte	Sensitizer	PCE (%)		Reference
			Front	Rear	
TeMC	$Co(bpy)_3^{3+/2+}$	SM315	9.43	8.06	This work
Carbon quantum dot	I^-/I^{3-}	N719	9.08	7.01	1
PEDOT/MWCNT	I^-/I^{3-}	N719	9.07	5.62	2
Carbon black	$Co(bpy)_3^{3+/2+}$	Z907	5.44	4.41	3
CuSe	I^-/I^{3-}	N719	7.22	5.38	4
Transparent carbon	I^-/I^{3-}	N719	6.07	5.04	5
SWNT	$Co(bpy)_3^{3+/2+}$	MK-2	4.81	4.56	6
CoSe	I^-/I^{3-}	N719	8.30	4.63	7
PEDOT	$Co(bpy)_3^{3+/2+}$	Y123	8.65	7.48	8
PEDOT	I^-/I^{3-}	N719	7.40	5.23	8

Supplementary References

[1] W. Zhu , Y. Zhao , J. Duan , Y. Duan , Q. Tang and B. He, Carbon quantum dot tailored counter electrode for 7.01%-rear efficiency in a bifacial dye-sensitized solar cell. *Chem. Commun.***2017**, 53, 9894, doi: 10.1039/c7cc05480g.

[2] H. Li, Y. Xiao, G. Han, Y. Zhang, A transparent honeycomb-like poly(3,4-ethylenedioxythiophene)/ multi-wall carbon nanotube counter electrode for bifacial dyesensitized solar cells. *Organic Electronics* **2017**, 50, 161-169, doi: 10.1016/j.orgel.2017.07.048.

[3] I.-P. Liu, Y.-C. Hou, C.-W. Li and Y.-L. Lee, Highly electrocatalytic counter electrodes based on carbon black for cobalt(III)/(II)-mediated dyesensitized solar cells. *J. Mater. Chem. A* **2017**, 5, 240-249, doi: 10.1039/c6ta08818j.

[4] P. Li, Q. Tang, Highly transparent metal selenide counter electrodes for bifacial dyesensitized solar cells. *J. Power Sources* **2016**, 317, 43-48, doi: 10.1016/j.jpowsour.2016.03.081.

[5] Bu, C.; Liu, Y.; Yu, Z.; You, S.; Huang, N.; Liang, L.; Zaho, X.-Z, Highly Transparent Carbon Counter Electrode Prepared via an in Situ Carbonization Method for Bifacial Dye-Sensitized Solar Cells. *ACS Appl. Mater. Interfaces* **2013**, 5, 7432-7438, doi: 10.1021/am4017472.
[6] S. H. Seo, M. H. Kim, E. J. Jeong, S. H. Yoon, H. C. Kang, S. I. Cha and D. Y. Lee, High electrocatalytic activity of low-loaded transparent carbon nanotube assemblies for CoII/IIImediated dye-sensitized solar cells. *J. Mater. Chem. A* **2014**, 2, 2592-2598, doi: 10.1039/c3ta13959j.
[7] Y. Y. Duan, Q. W. Tang, B. L. He, Z. Y. Zhao, L. Zhu and L. M. Yu, Bifacial dye-sensitized solar cells with transparent cobalt selenide alloy counter electrodes. *J. Power Sources* **2015**, 284, 349-354, doi: 10.1016/j.jpowsour.2015.03.045.
[8] Kang, J. S.; Kim, J.; Kim, J. Y.; Lee, M. J.; Kang, J.; Son, Y. J.; Jeong, J.; Park, S. H.; Ko, M. J.; Sung, Y. E., Highly Efficient Bifacial Dye-Sensitized Solar Cells Employing Polymeric Counter Electrodes. *ACS Appl. Mater. Interfaces* **2018**, 10, 8611-8620, doi: 10.1021/acsami.7b17815.
